# A cloud-based learning module for biomarker discovery

**DOI:** 10.1093/bib/bbae126

**Published:** 2024-07-23

**Authors:** Christopher L Hemme, Laura Beaudry, Zelaikha Yosufzai, Allen Kim, Daniel Pan, Ross Campbell, Marcia Price, Bongsup P Cho

**Affiliations:** Department of Biomedical and Pharmaceutical Sciences, College of Pharmacy, University of Rhode Island, Kingston RI, USA; Rhode Island IDeA Network of Biomedical Research Excellence (RI-INBRE); Google Cloud, Reston VA, USA; Health Data and AI, Deloitte Consulting LLP, Arlington VA, USA; Google Cloud, Reston VA, USA; Health Data and AI, Deloitte Consulting LLP, Arlington VA, USA; Health Data and AI, Deloitte Consulting LLP, Arlington VA, USA; Google Cloud, Reston VA, USA; Department of Biomedical and Pharmaceutical Sciences, College of Pharmacy, University of Rhode Island, Kingston RI, USA; Rhode Island IDeA Network of Biomedical Research Excellence (RI-INBRE)

**Keywords:** biomarkers, proteomics, cloud computing, machine learning

## Abstract

This manuscript describes the development of a resource module that is part of a learning platform named “NIGMS Sandbox for Cloud-based Learning” https://github.com/NIGMS/NIGMS-Sandbox. The overall genesis of the Sandbox is described in the editorial NIGMS Sandbox at the beginning of this Supplement. This module delivers learning materials on basic principles in biomarker discovery in an interactive format that uses appropriate cloud resources for data access and analyses. In collaboration with Google Cloud, Deloitte Consulting and NIGMS, the Rhode Island INBRE Molecular Informatics Core developed a cloud-based training module for biomarker discovery. The module consists of nine submodules covering various topics on biomarker discovery and assessment and is deployed on the Google Cloud Platform and available for public use through the NIGMS Sandbox. The submodules are written as a series of Jupyter Notebooks utilizing R and Bioconductor for biomarker and omics data analysis. The submodules cover the following topics: 1) introduction to biomarkers; 2) introduction to R data structures; 3) introduction to linear models; 4) introduction to exploratory analysis; 5) rat renal ischemia-reperfusion injury case study; (6) linear and logistic regression for comparison of quantitative biomarkers; 7) exploratory analysis of proteomics IRI data; 8) identification of IRI biomarkers from proteomic data; and 9) machine learning methods for biomarker discovery. Each notebook includes an in-line quiz for self-assessment on the submodule topic and an overview video is available on YouTube (https://www.youtube.com/watch?v=2-Q9Ax8EW84).

This manuscript describes the development of a resource module that is part of a learning platform named ``NIGMS Sandbox for Cloud-based Learning'' https://github.com/NIGMS/NIGMS-Sandbox. The overall genesis of the Sandbox is described in the editorial NIGMS Sandbox [1] at the beginning of this Supplement. This module delivers learning materials on the analysis of bulk and single-cell ATAC-seq data in an interactive format that uses appropriate cloud resources for data access and analyses.

## INTRODUCTION

Biomarker discovery is a well-known discipline in modern biomedical research. While most researchers understand conceptually what a biomarker is, they may lack a formal definition or have differing views on what constitutes a biomarker based on their scientific background and research interest. Several groups including the US National Institutes of Health (NIH), the US Food and Drug Administration (FDA) and the World Health Organization (WHO) have worked to provide formal definitions of biomarkers [2–5]. In the broadest sense, a biomarker is any biological entity indicating a state change. In biomedical research, biomarkers are commonly used to measure the efficacy of a drug treatment, to analyze physiological changes across tissues or between different cohorts, to analyze physiological changes across time, for early prognosis of disease, or to assess the relative variability associated with physiological or experimental covariates.

While the modern concept of biomarkers originated with the emergence of the field of molecular biology, the idea of observing or measuring a biological entity as an indication of a change of state (such as progression of a disease) has a long history in medicine. The molecular biology age allowed quantitative assessment of molecular biomarkers within a consistent and reproducible statistical framework. While any omic dataset can be used to identify biomarkers, the proteome is increasingly the target of experimental studies. As the translated subset of the transcriptome, the proteome better represents the active physiological state of the subject, and proteins are more likely to be targets of existing therapeutic treatments and future drug development. With the emergence of single cell, spatial and multiomics methodologies, it will soon be possible to measure all potential biomarkers at the single cell level in a 3D spatial context [6]. However, the amount of data generated and analyzed is immense, and often requires multi-disciplinary teams of experimentalists, clinicians and bioinformaticians to interpret it. These different groups will view biomarker data from vastly different perspectives, which can complicate data interpretation. Bioinformaticians typically take a bird’s-eye view of the data, focusing on systems of correlated features. Experimentalists are primarily interested in the subset of the data directly relevant to the hypothesis being tested or the system being studied. At the same time, clinicians are most interested in the utility of the results and how they can be translated into tools for measuring clinical outcomes. For example, a bioinformatician may construct a dataset that comprehensively identifies direct and downstream indirect effects of a disease or treatment, and which may provide systems level insights into the mechanisms of disease. A clinician in contrast is most interested in those biomarkers which can be translated into accurate, practical tests for the disease state. Biomarker tests that do not measurably improve on existing tests are of little practical utility to the clinician.

To help bridge the communication gap between these groups, the Molecular Informatics Core of the Rhode Island Institutional Development Award (IDeA) Network of Biomedical Research Excellence [7, 8] developed a cloud-based learning module for biomarker discovery for the NIGMS Sandbox platform. This module consists of Jupyter Notebooks running R and Bioconductor to analyze serum and proteomic data from a rat renal ischemia-reperfusion injury (IRI) model [9, 10]. Because the field of biomarker research is vast and complex, this module is designed to introduce new researchers fundamental concepts in biomarker discovery that will form the basis of more sophisticated techniques the researcher may encounter in their future work. Users will learn how to assess and compare serum biomarkers using linear and logistic regression, how to conduct exploratory analysis on proteomic datasets (heatmaps, principal components analysis, etc.), how to deal with outliers and batch effects in proteomics data, and how to conduct basic differential analysis of proteomics data. The module includes background information on R programming, linear models and exploratory analysis for users who require it. Finally, users are introduced to basic concepts in machine learning (ML) (decision trees, random forest, etc.) that are increasingly being used for biomarker discovery. The module complements other existing modules in the NIGMS Sandbox (proteomics, RNA-seq, single-cell ATAC-seq, multiomics). It is not intended to be a comprehensive study of biomarker discovery methods but can serve as the foundation for future biomarker discovery modules covering topics such as advanced regression methods, multiomics and advanced ML algorithms.

The dataset used for this module consists of serum biomarker and proteomic data from a rat renal IRI model made available by Dr. Nisanne Ghonem at the Department of Biomedical and Pharmaceutical Sciences, College of Pharmacy, University of Rhode Island [9, 10]. IRI is a common problem in organ transplantation. When an organ is removed from a donor, it is temporarily deprived of oxygen (ischemia), resulting in depletion of cellular ATP stores, inflammation, mitochondrial dysfunction and apoptosis. Upon reattachment of the organ in the new host, the exposure to oxygenated blood (reperfusion) leads to production of reactive oxygen species and cellular damage. A variety of factors including time between transplantation of chilling of the transplanted organ may limit the extent of IRI, but there is currently no approved pharmacological treatment to limit the extent of injury. Dr. Ghonem’s laboratory explores the use of the FDA-approved prostacyclin analog Treprostinil (©Remodulin) to mitigate IRI damage. To test the therapeutic effects of Treprostinol in a IRI model, male Sprague Dawley rats were assigned randomly to control, sham (surgical treatment with no induced IRI), IRI/placebo or IRI/trep-treated groups. The groups were subjected to 45 min of bilateral renal ischemia through clamping to restrict blood flow to the kidney. This was followed by reperfusion after set times (1–72 h). 100 ng/kg/min placebo or Treprostinil was administered subcutaneously by osmotic pump. Serum biomarker data including serum creatinine (SCr) and blood-urea nitrogen (BUN) were collected for each sample. Tissue samples were extracted to analyze changes to the proteome between the different groups. Full details of the experimental protocols can be found in the associated manuscripts [9–11].

## METHODS

### Setting up the environment and running the modules

The long-term goal of the NIGMS Sandbox is to provide a set of learning modules that are platform agnostic, meaning that they can be used in any cloud environment. At the time of this writing, the modules are only available on the Google Cloud Platform (GCP) and were developed in coorperation with Google and Deloitte. The module is a series of nine submodules implemented as Jupyter Notebooks. Users will be required to set up a GCP account associated with a billing account. First time users new to GCP may also take advantage of $300 in free cloud credits from Google for 90 days. Instructions for setting up the environment for using the modules can be found using the instructions in the NIH Cloud Readme (https://github.com/STRIDES/NIHCloudLabGCP). Briefly, users will spin-up a virtual machine in GCP’s Vertex AI system and clone the module materials from the associated GitHub repository (https://github.com/NIGMS/Analysis-of-Biomedical-Data-for-Biomarker-Discovery). The repository contains all the materials required to run the module, including notebooks, data, images and quizzes. Each notebook will be run R and any necessary R packages will be loaded within the notebooks. Further details can be found in the README.md file in the GitHub repository.

### Overall design architecture

**Figure 1:**
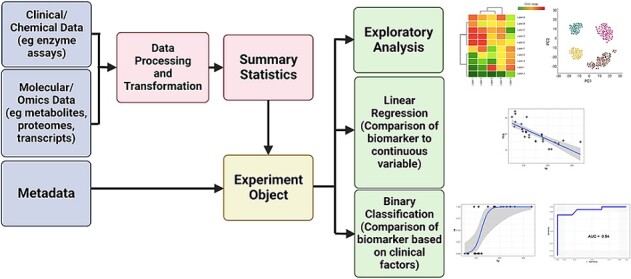
Architectural design of the biomarkers module. The first step in the module is collecting experimental data, metadata and reference information (as necessary) into an experimental object (e.g. an R list). Experimental data is processed to remove low-variability features and to normalize data across samples. The experimental object is then used for the subsequent analyses including exploratory analysis (PCA, identification of batch effects, heatmaps), regression analysis of clinical biomarkers and proteomics analysis. Figure created in BioRender.

The overall architecture design of the module is presented in Figure 1. Clinical biomarker data, proteomic data, and metadata are processed as needed (log transformation, normalization, etc.) and are collected into an R list that serves as an experimental object. This experimental object is then called upon and/or modified as the experimental analysis progresses. Each submodule is implemented as a Jupyter Notebook running R and Bioconductor. Each notebook utilizes in-line quizzes to allow for self-assessment of the topics covered in each submodule. A total of nine total submodules are available and are described below.

#### Submodules

**Figure 2:**
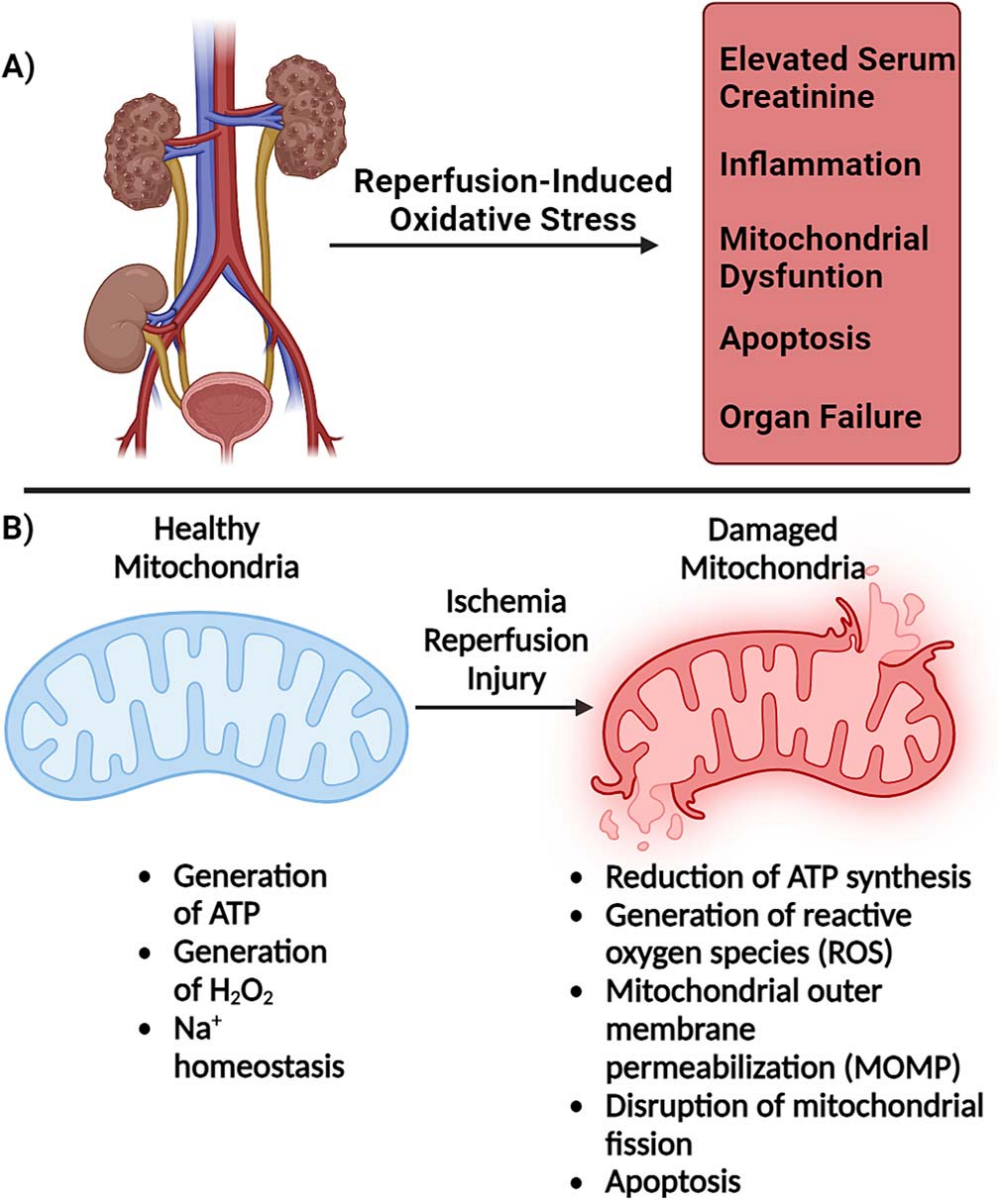
Rat renal IRI model. The model used in this module is the rat renal IRI model (**A**). When an organ is removed from a donor, it experiences a period of oxygen depletion (ischemia). This leads to inflammation, depletion of cellular ATP stores, mitochondrial dysfunction and apoptosis. Upon reattachment of the organ in the new host, the organ is exposed to oxygenated blood (reperfusion), which can lead to formation of reactive oxygen species, additional inflammation, and cell death (**B**). Treprostinil, a prostacyclin analog, is a potential treatment for mitigating IRI damage. Figure created in BioRender.

**Figure 3:**
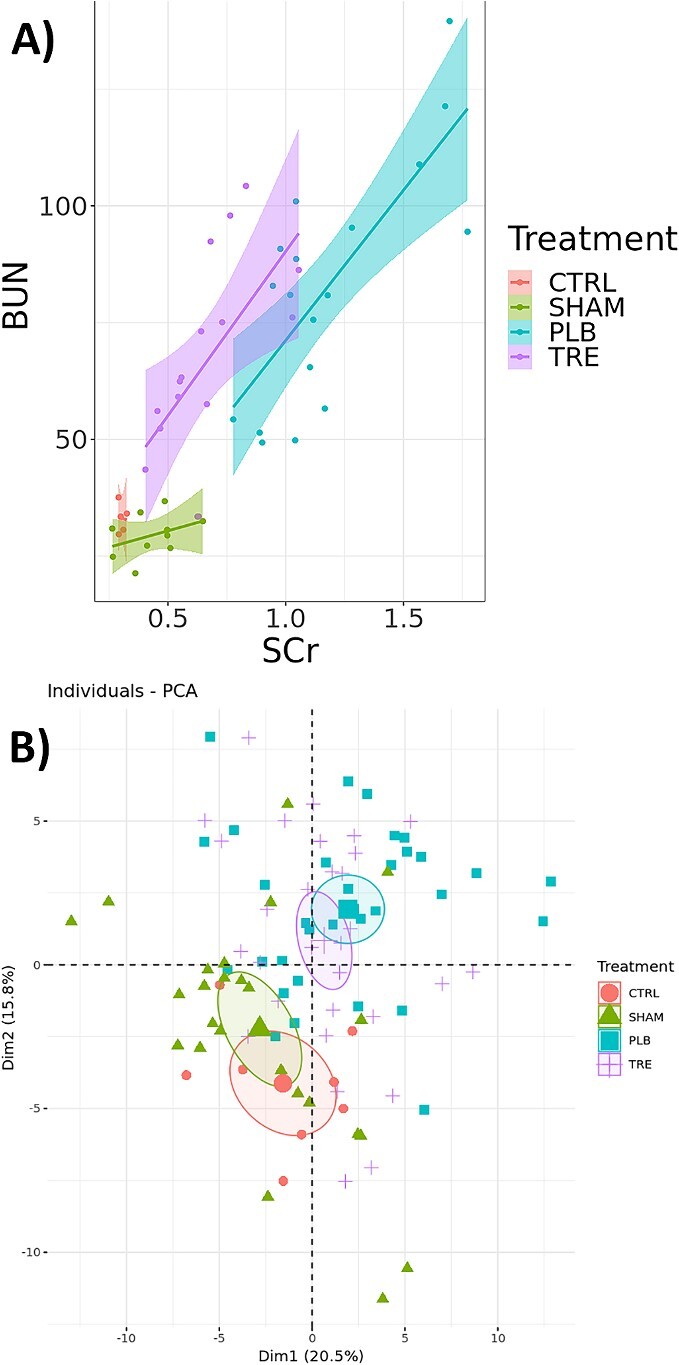
Examples of notebooks used in the module. The module is organized into nine submodules that cover a variety of topics in biomarker analysis. (**A**) Submodule 6 introduces concepts in regression models that can be applied to biomarker analysis. (**B**) Submodule 7 shows the user how to conduct exploratory analysis such as PCA on proteomics data.

**Figure 4:**
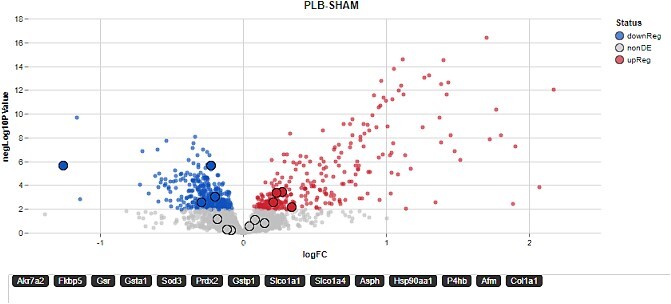
Volcano plot of IRI data generated in Glimma. A volcano plot generated in Submodule 8 (identification of IRI biomarkers from proteomic data) for sham versus placebo (Untreated IRI) at 24 h. The volcano plot was generated using the Glimma package that creates interactive plots. Proteins related to renal damage pathways are highlighted on the plot.

1) Introduction to biomarkers—this module provides a basic introduction to the concept of biomarkers. We examine properties of biomarkers that make them clinically relevant, such as specificity, sensitivity, accuracy, cost and clinical utility. This is followed by a discussion of different ways of classifying biomarkers. Biomarkers can be qualitative (presence of fever, jaundice, visible tumor) or quantitative (temperature, blood pressure) and can be observed on the macro- or molecular scales. The common blood test is used to demonstrate the types of molecular biomarkers that are routinely measured, followed by a discussion of omics-based biomarkers. More formal classifications of biomarkers are discussed (e.g. the Biomarkers, EndpointS and other Tools Glossary). Finally, various case studies covering different types of biomarkers are discussed (e.g. prostate-specific antigen, alkaline phosphatase activity, BReast CAncer gene variants, serum creatinine, microbial pathogens of periodontal disease).
2) Introduction to R data structures—for students needing additional background training, three optional submodules covering basic concepts are included. The first is a discussion of R data structures. Students are introduced to vectors, lists, data structures and matrices with an eye for how these data structures will be used in exploratory and regression analyses in later modules.
3) Introduction to linear models—the second optional submodule covers basic principles of linear algebra and linear models. The intent is to provide students with a foundation in the topics to prepare them for the submodules using applied linear models. Topics include vector and matrix operations, principles of linear models and an introduction to the generalized linear model with an emphasis on linear and logistic regression.
4) Introduction to exploratory analysis—the third optional submodule provides background on exploratory analyses used in later analysis of proteomic data. Specifically, this submodule covers the basics of principal components analysis, hierarchical clustering and k-means clustering. It also introduces students to the R tidyverse plotting philosophy used by the *ggplot2* package.
5) Rat renal IRI case study—with the fundamentals covered, Submodule 5 introduces the experimental system used for the remaining modules (Figure 2). The experimental data used are the rat renal IRI data that include serum biomarker data and proteomics data, and which is included with the module. Students are shown how to gather the data into an R list that serves as a central experimental object holding experimental and metadata.
6) Linear and logistic regression for comparison of quantitative biomarkers—Submodule 6 introduces students to linear and logistic regression methods using the serum biomarker data from Submodule 5 (Figure 3). Students are shown how to compare biomarkers using linear regression and how to use the metadata to define covariates. Biomarkers are also compared using logistic regression under different classification schemes and receiver operator characteristic (ROC) curves are used for evaluation.
7) Exploratory analysis of proteomics IRI data—in this submodule, students begin analyzing proteomic IRI data using PCA and heatmaps (Figure 3). Students are shown how to normalize data across samples, identify and correct for batch effects, remove low variability features, and identify potential signals in the data.
8) Identification of IRI biomarkers from proteomic data—Submodule 8 introduces differential analysis of proteomics data using the *limma* package in R Bioconductor. Students are shown how to correct for batch effects in a linear model, how to define contrasts in their model and how to visualize the results (Figure 4).
9) ML methods for biomarker discovery—the final submodule introduces basic concepts in ML and how they can be applied to biomarker discovery. Topics include defining test and training data sets, feature selection, and different ML models such as decision trees, random forest, gradient boosting machines and support vector machines.

No prerequisites are required to use this module aside from a basic understanding of molecular biology concepts (e.g. macromolecules, central dogma of biology, etc.). Submodule 1 provides the background in biomarkers, and all users should start there. Users who need additional background may work through Submodules 2–4 as required, otherwise, they may skip those modules and proceed to Submodule 5, which introduces the IRI system. Submodule 9 is not necessary to complete the module but is there for users interested in learning about fundamental concepts in ML and how ML can be applied to biomarker discovery.

## RESULTS

Beginning with Submodule 5, the user can access and manipulate the molecular IRI data. In general, regression analysis of omics data requires at minimum a count matrix of experimental values, metadata and a regression model. The count matrix is organized by sample (columns) and feature (rows), with each cell representing the value of that feature for that sample (e.g. # nucleotide reads, signal intensity, protein concentration, etc.). The metadata (organized as a tibble or data frame) includes phenotypic and other data about the samples, which allows for subsetting of the data and inclusion of covariates for the regression model. The regression model defines the covariates of interest and can be modified to test various hypotheses or to determine covariates of interest. A user wishing to utilize their own data using these methods would simply need to organize their data as a count matrix (while respecting R naming conventions for samples) and define the metadata and models needed. While most regression models in omics utilize a similar strategy, how the data are stored varies. In this module, the user creates an experimental object to hold the raw and processed experimental data and metadata about the samples. For the sake of simplicity, this object is a simple R list that is saved and reloaded into subsequent modules. An R list was chosen because it is intuitive for users new to R. Users are also briefly introduced to the Bioconductor Summarized Experiment object for future reference.

In Submodule 6, users begin manipulating serum biomarker data and metadata to learn basic concepts in linear and logistic regression. Users are shown how to make basic R summary plots such as scatter plots with regression lines and box plots. This dataset contains outliers that represent severe IRI damage that is likely resistant to any treatment, and which can be removed. The user is shown how to identify and remove these outliers. Linear regression is used to compare the relative effects of SCr and BUN concentrations based on treatment state and/or time. Common regression diagnostic plots (e.g. QQ plots, residuals plots, etc.) are generated and discussed. The users then learn how to use logistic regression to assess basic classification schemes and how to interpret logistic regression data compared to data from linear regression. ROC curves are generated using the R plotROC package [12], and users are shown how to interpret features of the ROC curve such as the area under the curve to evaluate the classification scheme. For the IRI data, users can see a linear relationship between SCr and BUN and that there are distinct differences in biomarker concentrations between the groups. Specifically, the placebo group has higher concentrations of each biomarker, indicating IRI, compared to the sham group, but treatment with Treprostinil can partially mitigate this damage. Logistic regression further shows that standard clinical cutoffs for SCr and BUN can be used to distinguish between the groups.

Submodule 7 focuses on exploratory analysis of proteomic data, primarily PCA and heat maps. The users are shown how to generate and plot PCA data using biplots. This dataset has a clear batch effect evident in the PCA plots, and so time is devoted to discussing batch effects and different ways to account for them. Users are also shown how to subset their data to only the most highly variable features to clarify the PCA results. Biplots are used to identify clusters of correlated features which are explored further using heatmaps using Bioconductor’s ComplexHeatmap package [13]. For the IRI data, the heatmaps and PCA show separation between the groups based on both treatment and time when limiting to the most highly variable proteins.

In Submodule 8, users perform differential analysis of the proteomics data. The limma package [14] of Bioconductor is used because of its status as the ‘grandfather’ package for omics analysis in R and provides a solid understanding of how regression-based differential analysis tools in general work. Users are shown how to build models that account for multiple covariates and batch effects, and how to define contrasts for pairwise analysis. Interactive diagnostic plots such as MA plots and volcano plots are built using the glimma package [15, 16] and their utility in evaluating proteomics data are discussed. Users can use different models and contrasts to see the effects of time and treatment on renal injury biomarkers in the IRI data set. The submodule concludes with ‘next step’ methods that often follow differential analysis such as pathway/gene set enrichment analysis, multiomics and meta-analysis. Interested users are also referred to the associated IRI manuscripts to get information on additional experimental analysis of the system [9–11].

## DISCUSSION

This module provides a basic vocabulary of biomarker discovery and assessment tools. It is intended to provide a foundation for concepts including data normalization, correction for outliers and batch effects, linear and logistic regression, exploratory analysis using PCA, differential analysis of omics data and fundamental ML models. The field of biomarker research is vast and sophisticated, so by necessity, this module does not cover every aspect of biomarker assessment. However, it is designed to complement existing and future modules in the NIGMS Sandbox. As of this writing, existing modules that complement the biomarker discovery module include RNA-seq, proteomics, single-cell ATAC-seq and multiomics (transcriptomics + epigenomics). Future modules that could build on this include advanced regression models for biomarker assessment, the use of multiomics and genome-wide association studies (GWAS) to enhance biomarker discovery, and advanced ML methods for biomarker discovery and evaluation.

The dataset used in this module is small enough that cloud computing is not necessary for analysis. However, biomedical datasets are becoming so large and complex that local analysis even on a high-performance computing cluster can be prohibitive. Three issues contribute to this problem. The first is simply the size of the data. Sequencing technology continues to advance, allowing for exponential growth of the datasets beyond the capacity of computing technology to keep up. As mass spectrometry technology has improved, the scale of proteomics and metabolomics data is beginning to show the same pattern. The second problem is the resolution of the data. Sample sizes of bulk omics experiments are typically limited by budget, but single cell omics technology can routinely generate data for tens of thousands (and eventually millions) of cells. The final problem is the complexity of the data. The statistical power of biomarker discovery can be increased by correlating multiple omics levels together along with GWAS and environmental data (multiomics). The prevalence of similar datasets allows for cross-comparison of omics data across experimental studies (meta-analysis) along with the associated host of batch effects.

These types of analyses are increasingly being conducted in the Cloud which gives several advantages including 1) easy access to large public databases (Gene Expression Omnibus, Sequence Read Archive, etc.), 2) remote, secure storage access for large datasets and 3) portability into cloud-based analysis workflows. Storing data such as public data repositories in cloud storage buckets reduces overhead and simplifies access to the data. Users can deploy their own bioinformatics pipelines (such as those created in Snakemake) in containers which can be deployed on the cloud or can utilize Cloud-based workflows such as NextFlow or Galaxy. Finally, the results can be directly ported into machine-learning-based analysis and visualization workflows implemented in the Cloud. Placing the data, users and tools in the same environment improves efficiency and reproducibility of workflows and simplifies data management.

In addition to the scientific content, this module gives users a basic introduction into cloud concepts such as storing data in and accessing data from cloud storage buckets, creating virtual machines, cloning remote repositories, etc. This and associated NIGMS Sandbox modules are the result of a productive collaboration between educational institutions, the Federal government, and the private sector. The long-term goal of the NIH is to move biomedical research to the cloud. These efforts are hampered by questions of cost, data security, privacy and a general lack of understanding of how the cloud works. These modules are designed to simplify the process of onboarding users to the cloud and to familiarize them with basic cloud concepts while also teaching practical skills in biomedical data science. Democratizing the use of computational resources is particularly important for institutions in IDeA states that often lack the funding and computational infrastructure required to do the most cutting-edge research. Furthermore, today’s students are graduating into an environment where traditional bulk omics methods are rapidly giving way to single cell/spatial multiomics studies utilizing ML and meta-analysis. This a paradigm shift in biomedical data science on par with the genomics revolution of the 1990s and cloud systems are increasingly required for these types of analyses. Students familiar with cloud systems will have a competitive advantage and will be well positioned to conduct the most cutting-edge research.

**Key Points**

- Provide users with an understanding of basic concepts in identifying and evaluating biomarkers
- Introduce concepts such as basic biomarker definitions and case studies
- Utilize a rat renal ischemia-reperfusion injury consisting of serum and proteomics to introduce concepts linear and logistic regression models exploratory and differential analysis of proteomics data, and basic ML concepts.
- Utilize in-line quizzes are used to provide self-assessment for the user.

## Data Availability

All module code and data sets used in the module are stored in a NIGMS Github repository (https://github.com/NIGMS/Analysis-of-Biomedical-Data-for-Biomarker-Discovery). A version of this module designed for Google Colab is also available (https://github.com/riinbre-bioinfo/Colab_Biomarkers).

## Notes

**Christopher Hemme** is the Director of the Rhode Island IDeA Network of Biomedical Research Excellence (RI-INBRE) Molecular Informatics Core (MIC). RI-INBRE operates out of the College of Pharmacy at the University of Rhode Island (URI) and consists of a network of nine Rhode Island universities The MIC provides bioinformatics and data science services to the RI-INBRE network.

**Laura Beaudry** is a program manager at Google. Her focus is on technical program management of engineering teams to ensure timely and impactful delivery of Google Cloud projects for Federal agencies and organizations.

**Zelaikha Yosufzai** is a Bioinformatics Analyst at Deloitte specializing in evaluating and manipulating bioinformatics data with software-based, statistical and data mining approaches.

**Allen Kim** is a Cloud AI/ML Engineer at Google. He is an experienced tech professional specializing in Machine Learning and Data Science.

**Daniel Pan** is a Cloud Architect at Deloitte Consulting. He is specializing in enhancing reliability and automation in Bioinformatics & LSHC Research.

**Ross Campbell** is a bioinformatician at Deloitte with a background in biomarker development from multi-omics experiments. Ross has expertise in optimizing bioinformatics machine learning pipelines to run at scale in cloud environments.

**Marcia Price** was a Cloud Program Manager at Google who executed program management, governance, and orchestration of large scale cloud engagements in the Federal Sector, particularly for the National Institute of Health.She is currently the Director, Training (Google Cloud Platform) at Jellyfish.

**Bongsup Cho** is the RI-INBRE Program Director and Pharmacy Professor at the University of Rhode Island College of Pharmacy.His research interests are the chemical biology of DNA damage and repair in the context of chemical carcinogenesis and cancer and 3D molecular science visualization.
